# A modified approach to percutaneous ultrasound-guided left stellate ganglion block for drug-refractory electrical storm: a case report

**DOI:** 10.1093/ehjcr/ytae101

**Published:** 2024-02-20

**Authors:** Francesco De Giorgi, Gaetano Scaramuzzo, Matteo Bertini, Michele Malagù

**Affiliations:** Anesthesia and Intensive Care Unit, Department of Translational Medicine, University of Ferrara, Ferrara, Italy; Anesthesia and Intensive Care Unit, Department of Translational Medicine, University of Ferrara, Ferrara, Italy; Cardiology Unit, Azienda Ospedaliero-Universitaria di Ferrara, Via Aldo Moro, 8, Ferrara 44124, Italy; Cardiology Unit, Azienda Ospedaliero-Universitaria di Ferrara, Via Aldo Moro, 8, Ferrara 44124, Italy

**Keywords:** Stellate ganglion block, Neuromodulation, Ultrasound, Electrical storm, Case report

## Abstract

**Background:**

The use of percutaneous stellate ganglion block (SGB) in the management of drug-refractory electrical storm (ES) has been increasingly reported in the last years. Few data are available on the safety, duration, and dosage of local anaesthetic used.

**Case summary:**

A 66-year-old male patient with a history of ischaemic cardiomyopathy and an implantable cardioverter–defibrillator (ICD) presented to the emergency room complaining several ventricular arrhythmias and ICD shocks received in the last 24 h. He was treated with many lines of anti-arrhythmic drugs but his condition deteriorated with cardiovascular instability and respiratory distress, so he was intubated. The ES still worsened (82 episodes of ventricular arrhythmias), so we performed an ultrasound-guided left SGB, using a modified technique, with success in suppressing the ventricular arrhythmias. The patient was then treated with electrophysiological study and catheter ablation.

**Discussion:**

The ultrasound approach to SGB is feasible in emergency setting, and it is safe and effective also using a modified and easier technique in patient with difficult sonographic visualization of the neck structures. Moreover, it is possible and safe to use a combination of short-acting rapid-onset local anaesthetic with a long-lasting one with a good outcome.

**Learning points**

- Stellate ganglion block is feasible in emergency setting, and it is safe and effective.
- Stellate ganglion block can be used as bridging therapy to catheter ablation for ventricular arrhythmias.
- Transverse process of C6 could be used as target for ultrasound-guided stellate ganglion block.
- Transverse process of C6 is easily identifiable with ultrasonography, even in case of difficult sonographic visualization of neck structures.

## Introduction

An electrical storm (ES) is defined as the occurrence of three or more episodes of ventricular tachyarrhythmias within a 24-h period, requiring treatment with anti-tachycardia pacing or implantable cardioverter–defibrillator (ICD) shocks in patients with ICD.^1^ While anti-arrhythmic drugs remain the primary treatment option, catheter ablation effectively manages ES but may not be feasible in emergency situations.^2^ Drug-refractory ES can result in haemodynamic deterioration and a high mortality rate.^3,4^ In such cases, neural modulation of the autonomic nervous system has been suggested to reduce ventricular arrhythmias and control ES in the acute phase.^5^

## Case presentation

**Figure 1:**
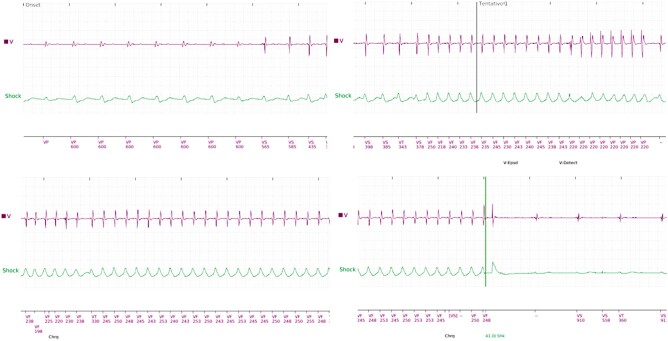
Ventricular tachycardia treated with anti-tachycardia pacing and shock. VF, ventricular fibrillation; VF, ventricular tachycardia.

A 66-year-old male patient presented to the emergency department with complaints of fever and ICD shocks. The patient had a history of heart failure, ischaemic cardiomyopathy (underwent previous coronary artery bypass graft and percutaneous coronary interventions), mildly reduced ejection fraction, and previous episodes of ventricular tachycardia (VT) and is an ICD recipient. Analysis of the ICD record revealed 10 episodes of VT/ventricular fibrillation (VF) within the last 24 h. Some VT/VF episodes resolved spontaneously, while others were interrupted by an ICD shock (*Figure 1*).

Consequently, the patient was admitted to the coronary intensive care unit. A chest X-ray indicated right basal pneumonia. Serum electrolytes were normal, but C-reactive protein was elevated (10.17 mg/dL). The patient received initial treatment with empirical antibiotics for the infection and anti-arrhythmic measures such as amiodarone and magnesium sulfate.

After admission, the patient experienced sustained VT recurrence with haemodynamic instability, unresponsive to anti-tachycardia pacing, and requiring multiple shocks from the ICD. Additional anti-arrhythmic drugs (lidocaine, procainamide, and beta-blockers) were administered, and sedation was initiated using midazolam and morphine.

A coronary angiography was performed to rule out an ischaemic trigger, but no coronary obstructions were found. After the procedure, VT/VF episodes persisted, and the patient developed respiratory distress and neurologic deterioration, necessitating orotracheal intubation, and mechanical ventilation.

A total of 82 VT/VF episodes and 81 ICD shocks were documented within 6 h of hospital admission. Due to the persistent anti-arrhythmic storm and the impossibility of performing an emergent electrophysiological study (EPS), a decision was made to conduct a left stellate ganglion block (SGB).^6^

The SGB was performed using a linear probe (7–12 MHz) and a 50 mm atraumatic needle.

**Figure 2:**
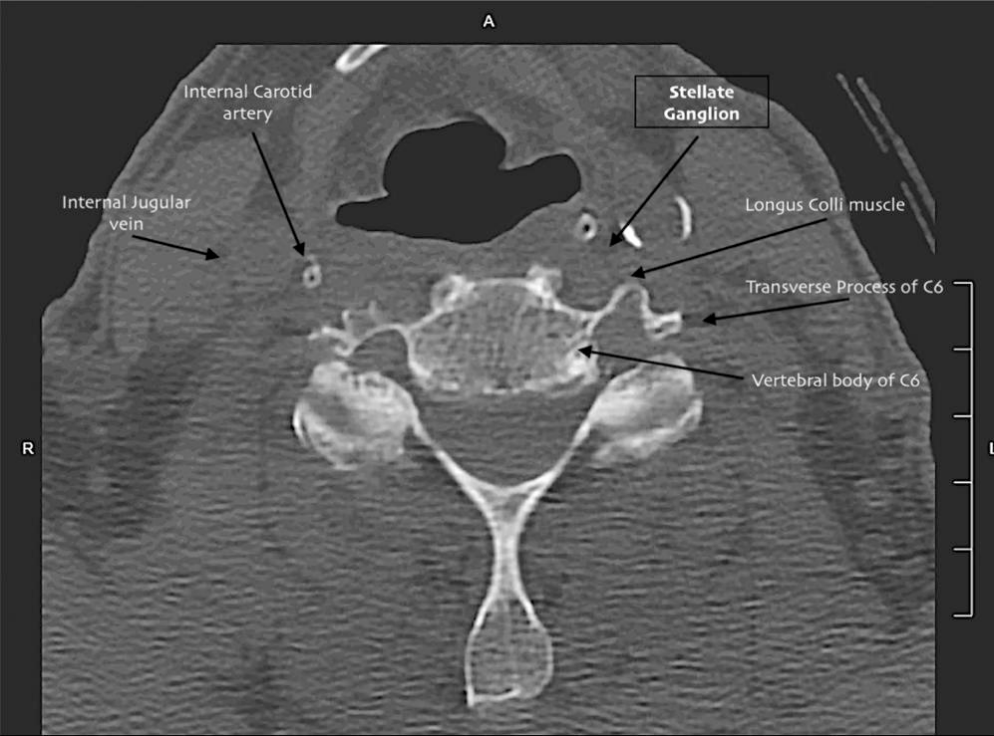
Computed tomography scan cross section of fascial layer of neck. The usual target of stellate ganglion block is the anterior plane of longus colli muscle. Our modified approach to stellate ganglion block takes as target the transverse process of vertebral bone C6.

Due to a challenging sonographic visualization of the neck structures (because of patient conformation), the lateral portion of the transverse process of C6 was used as a landmark target, deviating from the classical ultrasound approach that uses the *longus colli* muscle (*Figure 2*).

**Figure 3:**
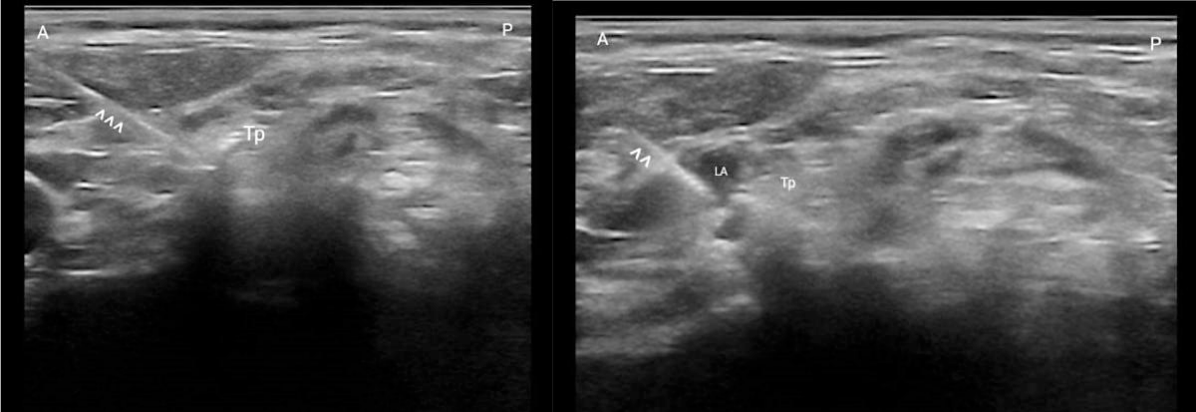
^^^, needle; LA, local anaesthetic; Tp, trasverse process.

The block was approached with an in-plane technique and anterolateral to posteromedial direction of the needle, aiming for contact with the bone. Once the transverse process was reached, following negative aspiration, lidocaine 2% (5 mL, 100 mg) and levobupivacaine 0.5% (8 mL, 40 mg) were injected, with the injection monitored under direct ultrasonography (*Figure 3*). This ratio of local anaesthetic would allow a rapid-onset (i.e. seconds to minutes) and long-lasting effect (i.e. some hours).

**Figure 4:**
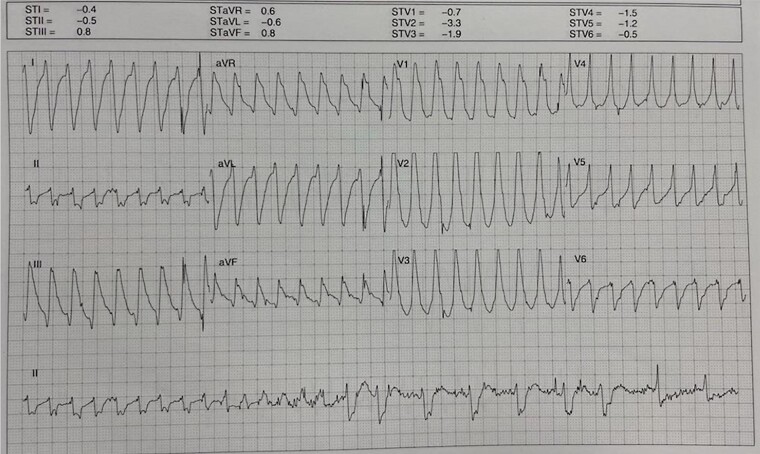
Twelve-lead electrocardiogram of the arrhythmia.

A few minutes after the SGB, complete arrhythmia suppression was observed, along with the restoration of sinus rhythm, improved haemodynamic parameters, and clinical stability. Two days later, an EPS revealed the inducibility of VT with a critical isthmus in the posterior basal region of the left ventricle (*Figure 4*). Transcatheter ablation was performed, resulting in arrhythmia interruption during ablation. Further substrate homogenization was performed. A few hours later, the patient was extubated and remained clinically stable with no further episodes of ventricular arrhythmias. At the 3-month follow-up, no additional arrhythmias were documented, and the patient was in good condition.

## Discussion

An ES is a rare yet life-threatening clinical scenario that can be addressed through a step-by-step approach, as suggested in recent literature.^7^

In our case, the initial step involved identifying and treating potentially reversible causes, such as infection, electrolyte imbalances, and ischaemia. Attempts were made with anti-arrhythmic drugs, but they proved ineffective as the ES remained refractory despite deep sedation. Due to the associated haemodynamic instability, an alternative strategy involving autonomic modulation was pursued.

The significance of the autonomic nervous system as a potential target for ventricular arrhythmias has gained prominence in recent years.^8^ Percutaneous SGB is perhaps the most recognized technique for temporary neuromodulation. One of the advantages of SGB is its theoretical feasibility even in emergency settings when other treatments, such as catheter ablation, may not be viable.

Stellate ganglion block can be performed using either an anatomical approach, as described by Walls *et al*.,^9^ or with the aid of ultrasonography. While the anatomical approach is quicker, it remains a ‘blind’ procedure with the potential risk of injuring neck structures (e.g. thyroid, carotid artery, and jugular vein). The ultrasound-guided approach requires more time but is considered safer and boasts a high success rate.

No specific guidelines exist for this procedure due to its rarity and the challenges of conducting randomized controlled trials in such scenarios. Limited information is available regarding the optimal drugs, dosages, volume, and duration of the SGB’s effects.

Recent case series^10,11^ have shed light on the efficacy and safety of both anatomical and ultrasound-guided SGB. In our case, given the unfavourable anatomy of our patient (i.e. a short and thick neck) and difficulties in identifying both anatomical and sonographic landmarks, we targeted the most lateral portion of the transverse process of C6. This area, away from neck vascular structures (e.g. carotid, thyroid, and vertebral arteries), is easily recognizable with ultrasound, enabling a rapid SGB in emergency scenarios.

In this innovative ‘hybrid’ approach, we utilized the classical landmark of the anatomical approach (i.e. the transverse process of C6) since the usual target for the ultrasound-guided block (i.e. the *longus colli* muscle) was not clearly distinguishable. Additionally, we used two different local anaesthetics to leverage the rapid onset of lidocaine and the long-lasting effect of levobupivacaine.

## Conclusions

The ultrasound approach to SGB is viable in emergency settings, proving to be both safe and effective when employing a modified technique that targets the transverse process of C6. This method is particularly advantageous in patients with challenging sonographic visualization of neck structures. The use of a short-acting, rapid-onset local anaesthetic in combination with a long-lasting one ensures sustained neuromodulation.

## Data Availability

The data underlying this article will be shared on reasonable request to the corresponding author.

## Lead author biography

Francesco De Giorgi is a medical doctor from the University of Ferrara currently completing his training in anaesthesia, intensive care, and pain medicine.

**Consent:** The authors confirm that written consent for submission and publication of the case report has been obtained from the patient in line with COPE guidance.

**Funding:** None declared.
